# Regenerative capacity in the lamprey spinal cord is not altered after a repeated transection

**DOI:** 10.1371/journal.pone.0204193

**Published:** 2019-01-30

**Authors:** Kendra L. Hanslik, Scott R. Allen, Tessa L. Harkenrider, Stephanie M. Fogerson, Eduardo Guadarrama, Jennifer R. Morgan

**Affiliations:** The Eugene Bell Center for Regenerative Biology and Tissue Engineering, Marine Biological Laboratory, Woods Hole, Massachusetts, United States of America; University of Sheffield, UNITED KINGDOM

## Abstract

The resilience of regeneration in vertebrates is not very well understood. Yet understanding if tissues can regenerate after repeated insults, and identifying limitations, is important for elucidating the underlying mechanisms of tissue plasticity. This is particularly challenging in tissues, such as the nervous system, which possess a large number of terminally differentiated cells and often exhibit limited regeneration in the first place. However, unlike mammals, which exhibit very limited regeneration of spinal cord tissues, many non-mammalian vertebrates, including lampreys, bony fishes, amphibians, and reptiles, regenerate their spinal cords and functionally recover even after a complete spinal cord transection. It is well established that lampreys undergo full functional recovery of swimming behaviors after a single spinal cord transection, which is accompanied by tissue repair at the lesion site, as well as axon and synapse regeneration. Here we begin to explore the resilience of spinal cord regeneration in lampreys after a second spinal transection (re-transection). We report that by all functional and anatomical measures tested, lampreys regenerate after spinal re-transection just as robustly as after single transections. Recovery of swimming, synapse and cytoskeletal distributions, axon regeneration, and neuronal survival were nearly identical after spinal transection or re-transection. Only minor differences in tissue repair at the lesion site were observed in re-transected spinal cords. Thus, regenerative potential in the lamprey spinal cord is largely unaffected by spinal re-transection, indicating a greater persistent regenerative potential than exists in some other highly regenerative models. These findings establish a new path for uncovering pro-regenerative targets that could be deployed in non-regenerative conditions.

## Introduction

High regenerative capacity has been demonstrated in a number of invertebrate and vertebrate animals. Classic models for whole body regeneration include hydras, planarians, and many annelids, which can regenerate entire animals with proper body form from tiny pieces of tissues including after repeated amputations [1–3]. Similarly, many instances of organ and tissue regeneration have been observed amongst vertebrate species. For example, zebrafish can regenerate complex tissues and organs including the heart, liver and fins [4, 5]. Mexican axolotl salamanders are known to regenerate their limbs, tails, skin, and several internal organs [6–13]. Other amphibians such as *Xenopus* tadpoles can regenerate spinal cord, limb buds, tail and lens [14, 15]. This regenerative capacity is not limited to non-mammalian vertebrates, as neonatal mice can regenerate digit tips and parts of their heart [16–18].

Remarkably, even tissues with a large number of terminally differentiated cells, such as the central nervous system (CNS, i.e. brain and spinal cord), can readily regenerate in vertebrates. As examples, zebrafish and amphibians can regenerate parts of their retina, optic nerve, and brain [19–25]. Species ranging from lampreys and bony fishes to amphibians and reptiles can regenerate spinal cord structures [19, 21, 26–29]. Although regeneration of the CNS is poor in mammals, peripheral nerve regeneration is particularly robust in most vertebrates, including adult mammals [30–32]. While these and many other examples of successful regeneration have been demonstrated across the animal kingdom for over a century, what is not understood is how well regenerative capacity persists after repeated injuries.

Repeated rounds of injury and recovery have been followed in only a small number of experimental models, with surprisingly varied outcomes on regenerative capacity. At one extreme, whole planarians can regenerate entire body structures from as little as 1/279^th^ of the original parent animal [33, 34]. Because planarians reproduce by fission, they can survive repeated rounds of resection and regeneration and are therefore technically immortal. Likewise, the zebrafish caudal fin can undergo repeated cycles of normal regeneration even after 27 amputations at the same location [35]. At the other extreme, salamanders and newts exhibit imperfect regeneration of limb structures beginning with the second amputation [36–38]. Therefore, regenerative capacity is limited in certain cases, and this may be due to distortion or displacement of tissues upon serial lesions, or atypical deposition of collagen-rich fibrotic scar tissue [36].

In comparison to the examples described above, very little if anything is known about how regeneration of nervous system tissues is affected by repeated injuries. Yet, understanding the extent of regenerative capacity in the spinal cord or brain could provide important insights into the mechanisms of nervous system plasticity, as well as the limitations that occur in higher vertebrates such as mammals. To begin testing the resilience of regenerative capacity in the vertebrate nervous system, we followed the behavioral and anatomical outcomes after two successive spinal cord transections in sea lampreys, *Petromyzon marinus*. Lampreys undergo robust functional recovery of swimming behaviors by 10–12 weeks after completely transecting, or severing, the spinal cord [39–43]. Spinal-transected lampreys gradually regain undulatory swimming behaviors to a degree that is qualitatively difficult to distinguish from the swimming behaviors of uninjured lampreys [39, 42], and kinematic analyses reveal only mild differences in swim speed, tail beat frequency, and body wavelength [41]. Behavioral recovery is accompanied by tissue repair at the lesion site, regeneration of descending and ascending axons several millimeters beyond the lesion site, and formation of new synaptic connections [41, 42, 44–47]. Amongst the descending neurons are 32 identified “giant” reticulospinal (RS) neurons, which reside in stereotypical locations in the lamprey midbrain and hindbrain, which have known probabilities of survival and regeneration. While some identified RS neurons are reproducibly “good survivors/regenerators,” others are “poor survivors/regenerators”, a unique feature of the lamprey model that allows for determination of regenerative capacity at the level of individual neurons [48–52]. In this study, we measured functional and anatomical recovery after an initial spinal cord transection and also after a second spinal transection (re-transection) at the same lesion site. We report here nearly identical behavioral recovery, synapse and cytoskeletal distributions, and neural regeneration after spinal transection and re-transection, with only minor differences in tissue repair, indicating that spinal cord regeneration in lampreys is resilient to repeated injuries.

## Materials and methods

### Spinal cord surgeries

Spinal cord transections were performed as previously described [41, 53, 54]. Briefly, late stage larval sea lampreys (*Petromyzon marinus*; 11–13 cm; 5–7 years old) were first anesthetized in 0.2 g/L MS-222 (Tricaine-S; Western Chemical, Inc.; Ferndale, WA). Once anesthetized, a small horizontal incision was made at the level of the 5^th^ gill through the skin and muscle to reveal the spinal cord, after which it was completely transected using fine iridectomy scissors. The spinal transection was visually confirmed, and the incision was subsequently closed with sutures (Ethilon 697G Ethilon Nylon Suture; Ethicon US, LLC; Somerville, NJ). Animals were housed post-operatively in isolated tank breeders within 10-gallon aquaria and held at room temperature (RT; 20–25°C). At 11 weeks post-injury (wpi), the regenerated lampreys were re-anesthetized, and their spinal cords were re-transected through the original lesion scar (also confirmed visually) using the same procedure. After spinal re-transection, the lampreys were allowed to recover for another 11 wpi prior to tissue harvest. All procedures were approved by the Institutional Animal Care and Use Committee at the Marine Biological Laboratory in Woods Hole, MA in accordance with standards set by the National Institutes of Health.

### Behavioral analysis

After spinal transection or re-transection, the lampreys’ swimming movements were scored twice per week during the recovery periods, as previously described [41, 55]. The scoring criteria were as follows: 0 –immediately post-operatively, lampreys exhibited no response to a light tail pinch; 1 –only head movements were observed; no tail movements occurred below the lesion site; 2 –brief periods of self-initiated swimming occurred, but with atypical movements and body shapes; 3 –lampreys demonstrated longer periods of swimming with more normal undulations and fewer abnormalities; 4 –lampreys exhibited persistent bouts of swimming with normal sinusoidal undulations that were comparable to uninjured, control lampreys. For practical reasons, we followed two smaller cohorts of n = 8 and n = 10 lampreys through the two successive behavioral recovery periods after spinal transection and re-transection, and we observed no significant differences between them. Thus, the average movement scores and standard deviations were calculated for the entire cohort of n = 18 animals and graphed in GraphPad Prism 8.0.0 (GraphPad Software, Inc.; La Jolla, CA). Additionally, at 1, 3, and 11 wpi, during both recovery periods, still images of the lampreys’ movements were extracted from videos acquired using a Sony Handycam HDR-CX455. At 11 wpi after re-transection, the lampreys’ brains and spinal cords were dissected for further experimentation in the anatomical studies, as described below.

### Spinal cord dissection and bright field imaging

At the appropriate post-injury time points, lampreys were re-anesthetized, and a 4-cm length of the spinal cords surrounding the lesion site were microdissected in fresh, oxygenated lamprey Ringer: 100 mM NaCl, 2.1 mM KCl, 1.8 mM MgCl_2_, 4 mM glucose, 2 mM HEPES, 0.5 mM L-glutamine, 2.6 mM CaCl_2_, pH 7.4. For most experiments, the spinal cords were fixed immediately in 4% paraformaldehyde (PFA) in 0.1 M phosphate buffered saline (PBS, pH 7.4) for 3 hours at RT and then overnight at 4°C, followed by washing for 3 x 5 min with 0.1 M PBS (pH 7.4). Bright field images of fixed, unstained whole mounted lamprey spinal cords (at 1, 3, and 11 wpi after the spinal transection or re-transection) were acquired at 30X magnification using a Zeiss Axiocam503 color camera mounted to a Zeiss Axio Zoom.V16 fluorescence stereo zoom microscope (1X, 0.25 NA Zeiss Plan-NeoFluar Z objective). From these images, the gaps between the proximal and distal spinal cord stumps (at 1wpi Trans and Re-Trans) were measured at the level of the central canal using ImageJ/FIJI software, and the resulting data were analyzed using an unpaired Student’s t-Test in GraphPad Prism 8.0.0 (GraphPad Software, Inc.; LaJolla, CA). Similarly, the lesion-to-end ratio was determined at 3 and 11 wpi by dividing the width of the spinal cord at the lesion center by the mean width of the ends (measured at 3 mm proximal and distal to the lesion center), and the resulting data were graphed and analyzed using ANOVA statistics in Prism 8.0.0.

### Immunofluorescence assays

Next, fixed spinal cords were cryoprotected in 12%, 15%, and 18% sucrose in 0.1 M PBS, pH 7.4 for ≥ 3 hours each at RT, or overnight at 4°C. A 2-cm length of each spinal cord was then embedded horizontally in O.C.T. Compound (EM Sciences; Hatfield, PA). Horizontal sections (14 μm thickness) were collected onto Superfrost Plus microscope slides (Fisher Scientific; Pittsburgh, PA) using a Leica CM1850 cryostat and stored at -20°C until use.

Spinal cord sections taken through the center of the central canal were selected for immunostaining and further analysis because they contain predominantly motor tracts that mediate the swimming behaviors. Cryosections were incubated in blocking buffer containing 9.5% normal goat serum (Life Technologies, Carlsbad, CA) and 0.5% Triton-X 100 for 45 minutes at RT. Next, the sections were incubated in primary antibodies diluted at 1:100 in an antibody signal enhancer solution for 2 hours at RT, as described in [56]. The primary antibodies used for this study were: a mouse monoclonal antibody raised against lamprey neurofilament-180 (LCM16; kind gift from Dr. Michael Selzer); a mouse monoclonal SV2 antibody that was deposited to the DSHB by Dr. Kathleen Buckley (DSHB; Iowa City, IA) [57]; and a mouse monoclonal anti-α-Tubulin antibody (clone DM1A; Sigma-Aldrich; St. Louis, MO). The NF-180 antibody [58] and SV2 antibody [41, 49, 54, 59, 60] have been extensively characterized in lamprey nervous tissues. The α-tubulin antibody is further characterized here. After primary antibody incubations, the sections were washed for 3 x 10 minutes at RT in wash buffer (20 mM Na phosphate buffer pH 7.4, 0.3% Triton X-100, 450 mM NaCl), followed by a 1-hour incubation at RT in secondary antibody (1:300 Alexa Fluor 488-conjugated goat anti-mouse IgG (H+L); ThermoFisher Scientific). For labeling of actin cytoskeleton, sections were directly labeled with Acti-Stain 488 phalloidin (Cytoskeleton, Inc.; Denver, CO) at 1:200 diluted in blocking buffer for 45 min at RT. Finally, sections were washed in wash buffer for 3 x 5 min, followed by 15 min in 5 mM Na phosphate buffer, pH 7.4. Slides were then mounted with ProLong Gold antifade reagent with DAPI (Life Technologies, Inc.) in order to label nuclei. DAPI robustly labels densely packed nuclei of the ependymal cells, which form the central canal, thus confirming the section plane of interest. After immunostaining, sections were imaged in ZEN 2.3 software using a Zeiss Axiocam 503 color camera mounted onto a Zeiss Axio Imager.M2 upright microscope (10X, 0.3 NA and 40X, 1.3 NA Zeiss EC Plan-Neofluar objectives). From these images, we used ImageJ/FIJI to quantify the number of NF-180 (+) axons, as well as the mean fluorescence intensity for SV2, tubulin, and phalloidin, within the proximal, lesion, and distal regions of transected and re-transected spinal cords (n = 3–4 spinal cords/condition). Resulting data were then graphed and analyzed using ANOVA statistics in Prism 8.0.0.

### Anterograde labeling of regenerated axons

For a subset of experiments, bulk anterograde labeling of regenerated axons was performed in transected (n = 14) and re-transected (n = 7) spinal cords, as previously described (Lau et al., 2013). Briefly, axons were labeled with a fluorescent dye (5 mM Alexa Fluor 488-conjugated dextran; 10 kDa; Thermo Fisher, Inc. Waltham, MA), diluted in lamprey internal solution (180 mM KCl, 10 mM HEPES, pH 7.4) via a 1x1x1 mm piece of Gelfoam (Pfizer; New York, NY), which was applied 5 mm rostral to the lesion site. After application, the dye was allowed to transport for 3–6 days before harvesting the spinal cords. Labeled spinal cords were imaged live in lamprey Ringer. Confocal Z-stacks were collected using a Zeiss LSM 510 laser scanning confocal attached to a Zeiss Axioskop 2FS upright microscope (10X, 0.3 NA Zeiss Plan-NEOFLUAR objective). Maximum intensity projections of the spinal cords, ranging from 2 mm proximal to 5 mm distal to the lesion center, were generated using the Zeiss LSM software. After stitching the projections together in Adobe Photoshop, the number of labeled, regenerated axons was counted at 1 mm intervals starting from the center of the lesion using ImageJ/FIJI. Resulting data were then analyzed using ANOVA statistics in Prism 8.0.0.

### Retrograde labeling of regenerated neurons

Regenerated giant RS neurons were retrogradely labeled, as previously described [52]. Briefly, at 11 wpi, the regenerated axons in the transected and re-transected spinal cords were labeled by inserting a 1x1x1 mm pledget of Gelfoam soaked in 10 mM tetramethylrhodamine dextran (TMR-DA; 10 kDa; ThermoFisher) into the spinal cord at a location 5 mm caudal to the lesion site (Shifman, et al., 2008). The TMR-DA was allowed to retrogradely transport for 9 days prior to harvesting the brains. Brains were imaged live at 25-30x magnification using a Zeiss Axio Zoom.V16 fluorescence stereo zoom microscope. In order to determine which neurons had regenerated, the average (background-subtracted) fluorescence intensity for each RS neuron was measured using ImageJ/FIJI software. Such measurements allowed us to determine whether the neuron was truly labeled and not just auto-fluorescent, for example, and to avoid any experimenter bias in this determination. Giant RS neurons that had regenerated their axons distal to the lesion were identified by their fluorescently labeled cell bodies (i.e. positive mean fluorescence values), while giant RS neurons that did not regenerate were devoid of dye (i.e. zero or negative mean fluorescence values). The percentage of regenerated RS neurons was calculated for individual RS neuron types, whole brains, and subpopulations of “good regenerators” and “poor regenerators. Cell-by-cell data represent the mean percentage of regeneration for each RS neuron type calculated from a larger population of lamprey brains (n = 9–10) for each experimental condition. Since each brain possesses only 2 neurons of each type, it is difficult to calculate the standard error of regeneration per cell type per brain, and therefore we represented the mean population data, as in previous studies [49, 51–53]. However, the other data presented were calculated from larger groups of neurons (n>100 neurons/condition, 9–10 lamprey brains), and therefore represent mean per brain and standard deviation. The resulting data were analyzed using Student’s t-Test in Prism 8.0.0.

### Nissl staining

After imaging the regenerated neurons, the lamprey brains were subsequently fixed overnight at 4°C in 4% PFA in 0.1M PBS, pH 7.4, washed 3 x 15 min with 0.1M PBS (pH 7.4) and stained with Toluidine Blue O (EM Sciences; Hatfield, PA) to label the Nissl substance [49, 52]. Brains were incubated in 1% Toluidine Blue O solution containing 1% borax (pH 7.6) for 20 minutes at 37°C. The brains were then destained in Bodian’s fixative (72% EtOH; 5% glacial acetic acid; 5% formalin) until the desired tissue contrast was obtained. Next, the brains were dehydrated in 95% and 100% ethanol (2 x 5 min each) and cleared in cedarwood oil at 65°C for 2 hours prior to mounting on slides with Permount. Bright field images of whole brains and giant RS neurons were acquired at 20-80x magnification using a Zeiss Axio Zoom.V16 fluorescence stereo zoom microscope.

Image analysis was performed using ImageJ/FIJI software. The mean intensity of each giant neuron was measured. Cells with a positive mean intensity after background subtraction were labeled Nissl (+), and cells with a negative mean intensity were labeled Nissl (-). The average percentage of Nissl (+) cells was plotted by cell type, for the overall brain, and subpopulations of “good survivors” and “poor survivors” from n = 9–10 lampreys/condition. Statistical analyses were performed as described above for regenerated neurons. We also compared the relationship between regeneration and neuronal survival (i.e. Nissl +) for each identified RS neuron after spinal transection and re-transection. These data were best fit by positive linear regressions and were statistically compared with a One-Way Analysis of Covariance (ANCOVA) using the VassarStats website (http://vassarstats.net/ancova2L.html).

### Statistical analyses

Except where noted above, all means and standard deviations were calculated, statistical analyses performed, and graphing conducting using GraphPad Prism 8.0.0. The experimental sample sizes (n’s) were determined using a biostatistics program: http://www.quantitativeskills.com/sisa/calculations/samsize.htm. All datasets reported here were tested for a normal Gaussian distribution using Shapiro-Wilk and D’Agostino & Pearson normality tests in Prism 8.0.0. All datasets passed the Shapiro-Wilk test (i.e. p-value was “not significant”). In addition, the vast majority of datasets also passed the D’Agostino & Pearson test, except in a few instances where the datasets were simply too small to run the test. Statistical outlier tests were also performed on all datasets in Prism 8.0.0, and no outliers were identified. The individual data points behind means and variance measurements for the quantitative analyses presented in the figures are available in S1 Appendix.

## Results

### Lampreys exhibit normal functional recovery after spinal re-transection

**Fig 1:**
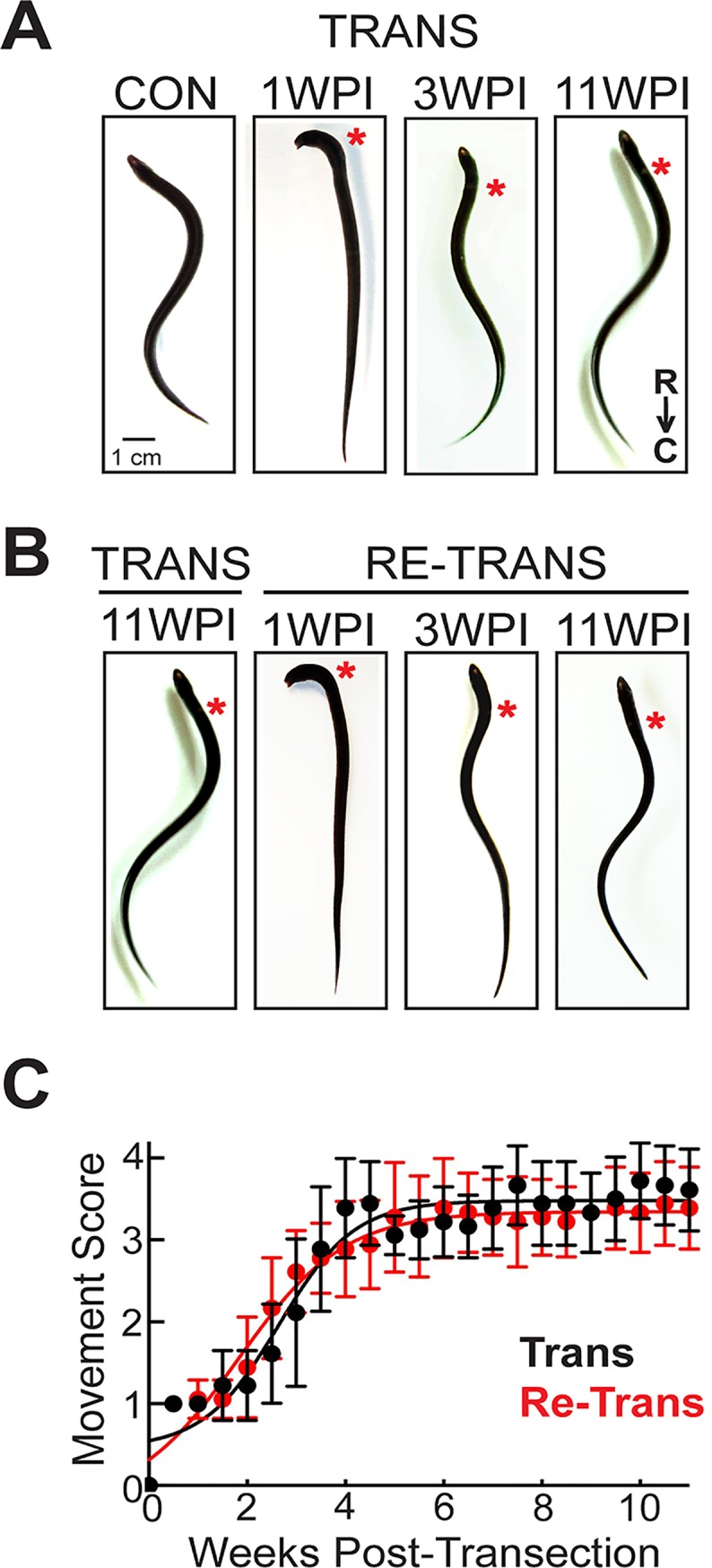
Normal functional recovery of swimming in lampreys after two successive spinal cord transections. **(A-B)** Still images of lampreys showing several stages of functional recovery after spinal transection (A) or re-transection (B). The body shapes are similar at each post-injury time point. Scale bar in panel A also applies to B. **(C)** Time course of functional recovery of swimming movements in transected vs. re-transected lampreys shows no difference (ANOVA, p = 0.37). Data points represent mean ± SD from n = 18 lampreys. Data were fit by a sigmoidal curve (Trans R^2^ = 0.95; Re-Trans R^2^ = 0.98).

The goal of this study was to determine the extent to which lampreys can functionally recover and regenerate their spinal cord structures after repeated injuries. We thus began by following the behavioral recovery after two successive rounds of injury (spinal transection and re-transection) in the same cohort of 18 lampreys. First, the lampreys were spinally transected at the level of the 5^th^ gill, after which they were allowed to recover for 11 weeks post-injury (wpi). At 1 wpi, the lampreys were paralyzed below the lesion site, and only head movements were observed (Fig 1A). At 3 wpi, the lampreys regained their ability to swim but displayed abnormal movements such as rapid head oscillations, abnormal body contractions, and shallow sinusoidal waves (Fig 1A). Once the lampreys reached 11 wpi, they exhibited normal undulatory, sinusoidal swimming movements that were similar to those of uninjured, control lampreys (Fig 1A). After this initial recovery period, the same lampreys underwent a second spinal transection at the original lesion site and were subsequently allowed to recover for another 11 wpi. Re-transected lampreys recovered along the same timeline and displayed similar locomotor behaviors (Fig 1B). During both recovery periods, the swimming behaviors were recorded twice per week using an observational movement scoring, as described in [41], where a score of 0 indicates complete paralysis; 1 indicates head wagging, but no forward movement; 2 indicates brief bouts of abnormal, self-initiated swimming; 3 indicates longer durations of persistent swimming with more regular movements; 4 represents normal sinusoidal swimming. The movement scoring indicated that both transected and re-transected lampreys recovered robustly along similar trajectories. After the initial spinal transection, the lampreys recovered to 90% of normal levels by 11 wpi (Fig 1C). The recovery process was best fit by a sigmoidal curve (Boltzmann) that reached a half maximum at 2.6 ± 0.2 wpi (Fig 1C) (R^2^ = 0.95, n = 18 lampreys), which was similar to previous reports [41, 55]. After spinal re-transection, this same cohort of lampreys recovered to 85% of normal swimming movements by 11 wpi, reaching half maximum at 2.4 ± 0.1 wpi (R^2^ = 0.98, n = 18 lampreys) with no significant difference from the initial recovery period (Two-way ANOVA, p = 0.37) (Fig 1C). Thus, remarkably, lampreys were able to recover normal swimming movements to the same degree after two consecutive spinal transections.

### Lesion repair is mildly altered but complete after spinal re-transection

**Fig 2:**
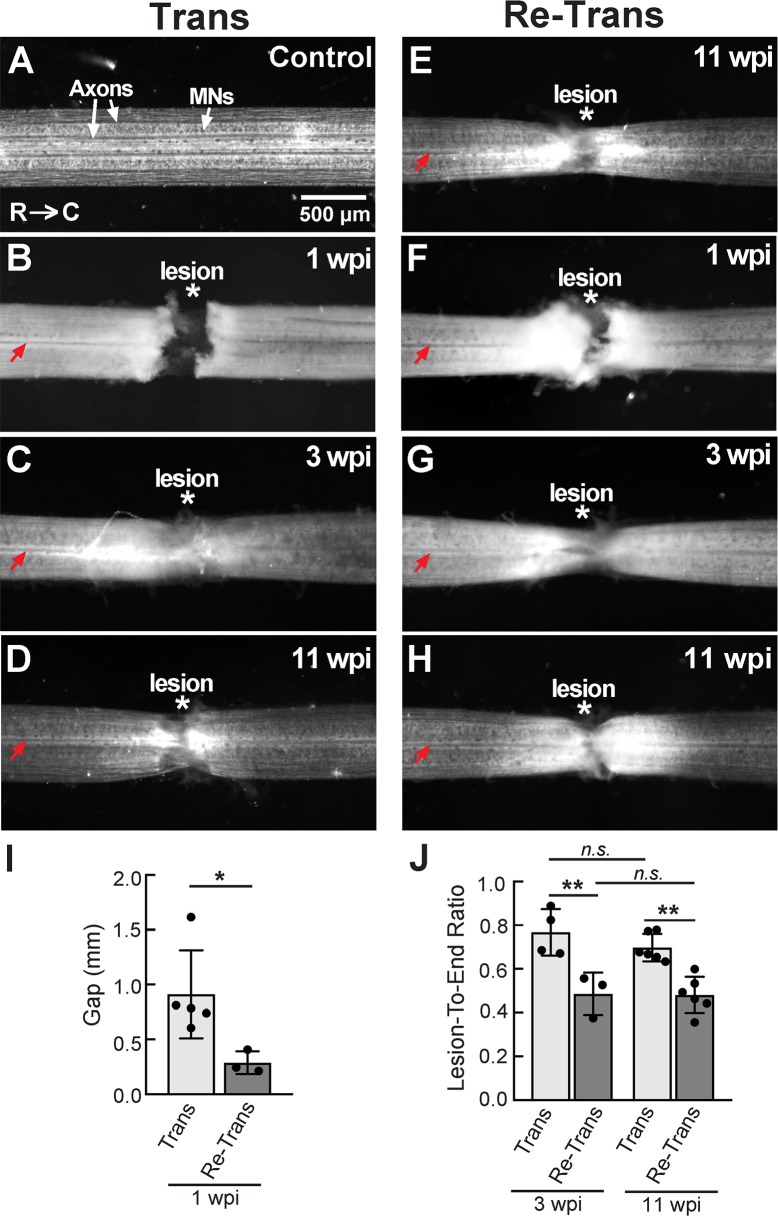
Robust tissue repair after spinal cord transection and re-transection. **(A-D)** Bright field images showing a fixed, unstained control lamprey spinal cord (A) and transected spinal cords (B-D) at the indicated time points. Axons and motor neurons (MNs) are clearly visible within the control spinal cord. At 1 wpi the proximal and distal stumps are still separated by a sizeable gap. But by 3–11 wpi, the spinal cord stumps are rejoined by extensive tissue repair. **(E-H)** Images showing the typical time course of lesion repair within re-transected spinal cords. Asterisks indicate the lesion center. Red arrows indicate the swollen central canal. R = rostral; C = caudal. Rostral is to the left in all images. Scale bar in A applies to B-H. **(I)** At 1 wpi, re-transected spinal cords exhibit a smaller gap between the proximal and distal stumps. Bars represent mean ± SD for n = 3–5 spinal cords. * indicates p<0.05 by unpaired Student’s t-Test. **(J)** At 3 and 11 wpi, the lesion-to-end ratios are smaller in re-transected spinal cords. Bars represent mean ± SD for n = 3–6 spinal cords. ** indicates p<0.005. *n*.*s*. = not significant by ANOVA.

Next, we examined the extent of tissue repair in the lamprey spinal cord after transection and re-transection. To do so, we performed bright field imaging on fixed, unstained lamprey spinal cords. Uninjured, control spinal cords are translucent and well organized with several giant reticulospinal axons and large spinal neurons (motor neurons and interneurons) visible along the longitudinal axis (Fig 2A). At 1 wpi after the initial spinal transection, the proximal and distal stumps of the spinal cord were largely disconnected, joined only by a thin layer of meninges that spanned the gap, and the central canal (red arrow) was swollen (Fig 2B and S1 Fig). At 3 wpi, the proximal and distal stumps had become re-connected by the re-formation of new spinal cord tissue that bridged the proximal and distal stumps at the lesion (Fig 2C and S2 Fig). This newly remodeled tissue was previously described to comprise longitudinally oriented glial and neural processes, as well as ependymal cells forming the new central canal [43, 61]. The central canal could be seen extending through the lesion center, still swollen. By 11 wpi, the tissue regained a more normal translucent appearance, and the lesion scar appeared more healed, though the central canal remained swollen (Fig 2D and 2E and S3 Fig). After spinal re-transection, the spinal cords generally exhibited similar gross morphologies at the same post-injury time points (Fig 2F–2H and S1–S3 Figs). A notable exception was at 1 wpi, where the re-transected spinal cords routinely exhibited advanced tissue repair at the lesion site, as demonstrated by a reduced gap between the proximal and distal stumps (Fig 2F and S1 Fig). Another interpretation is that the spinal cord stumps may have undergone less retraction after spinal re-transection, but this would also have the net effect of improving lesion repair. Corroborating this observation, at 1 wpi, the gap between the proximal and distal stumps was significantly smaller in re-transected spinal cords (Fig 2I and S1 Fig) (Trans: 0.91 ± 0.40 mm, n = 5 spinal cords; Re-Trans: 0.29 ± 0.10 mm, n = 3 spinal cords; Student’s t-Test, p = 0.04). Re-transected spinal cords were also narrower at the lesion site at both 3 and 11 wpi (compare Fig 2C and 2D and Fig 2G and 2H; S2 and S3 Figs). This was corroborated by a significant reduction in the lesion-to-end ratio (see Methods) (Trans 3 wpi: 0.77 ± 0.11, n = 4 spinal cords; Re-Trans 3 wpi: 0.49 ± 0.10, n = 3 spinal cords; Trans 11 wpi: 0.70 ± 0.06, n = 6 spinal cords; Re-Trans 11 wpi: 0.48 ± 0.08, n = 6 spinal cords; One-way ANOVA, p = 0.0002). Thus, in addition to behavioral recovery, there was also robust repair of the spinal cord tissues in re-transected lampreys, which occurred along a similar time course but with somewhat different anatomical characteristics.

### Axon, synapse, and cytoskeletal distributions are comparable after spinal transection and re-transection

**Fig 3:**
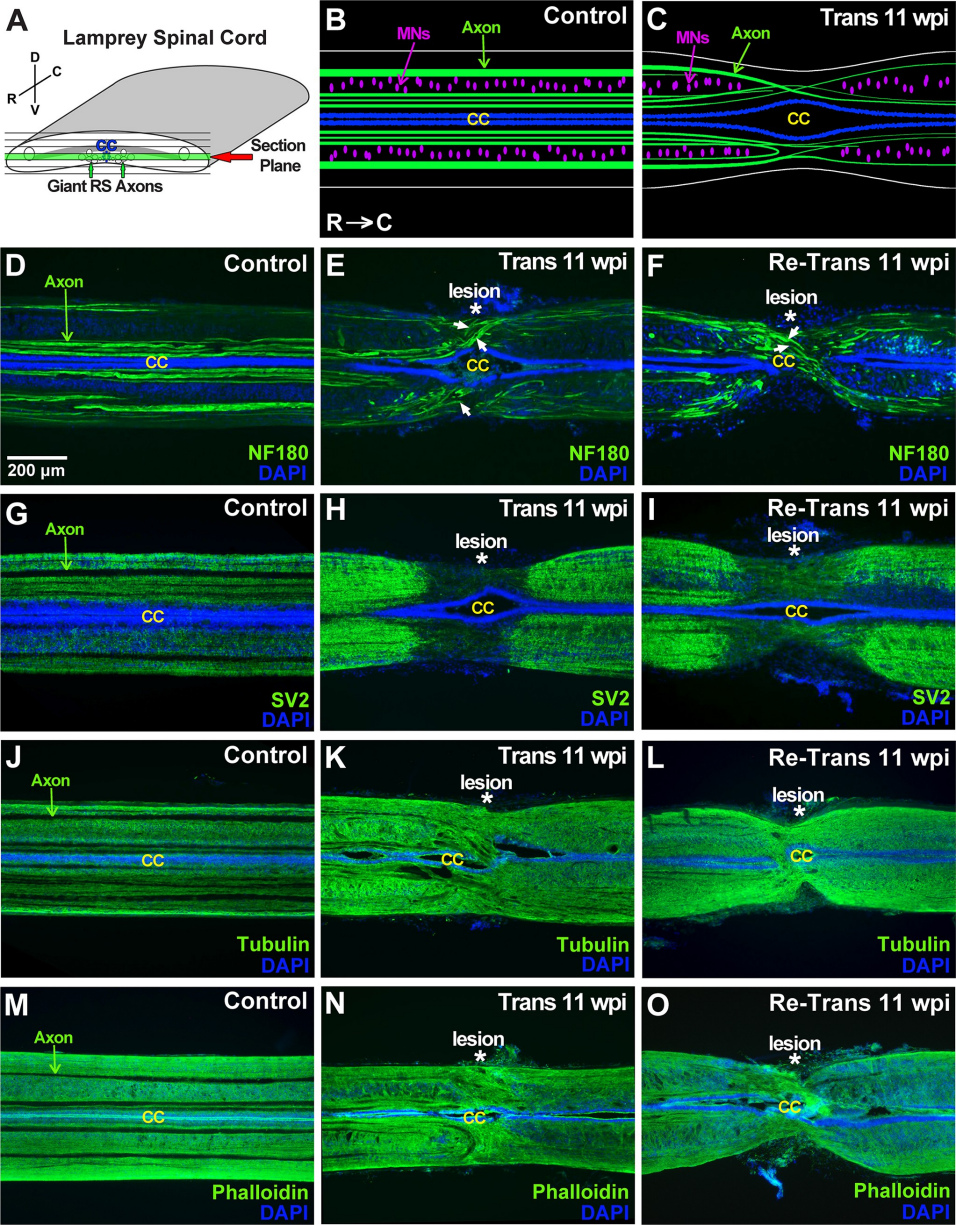
Distributions of axons, synapses, and cytoskeleton are similar at 11 wpi after spinal transection and re-transection. **(A)** Diagram showing the sectioning strategy and plane of interest. **(B-C)** Diagrams showing the basic anatomical features of uninjured control and transected (11 wpi) spinal cords. R = rostral; C = caudal. **(D-F)** NF-180 immunolabeling of control spinal cords shows large RS axons projecting in straight paths; DAPI labeling stains nuclei of ependymal cells that form the central canal (CC) and nuclei of intraspinal neurons. At 11 wpi in both transected and re-transected spinal cords, NF180-labeled axons project in aberrant patterns, and the CC is swollen. Asterisks indicate lesion center. Arrows indicate several regenerating axons crossing through the lesion. (**G-I)** SV2 immunostaining, which labels synapses, shows a uniform punctate pattern throughout the control spinal cord. After spinal transection and re-transection (11 wpi), SV2 staining is reduced within the lesion site. (**J-O)** Tubulin and phalloidin staining reveal relatively uniform microtubule and actin distribution, respectively, throughout control, transected and re-transected spinal cords, except in giant RS axons and the CC, which show reduced signal. No obvious differences were observed between transected and re-transected spinal cords. Rostral is to the left in all images. Scale bar in panel D also applies to E-O.

**Fig 4:**
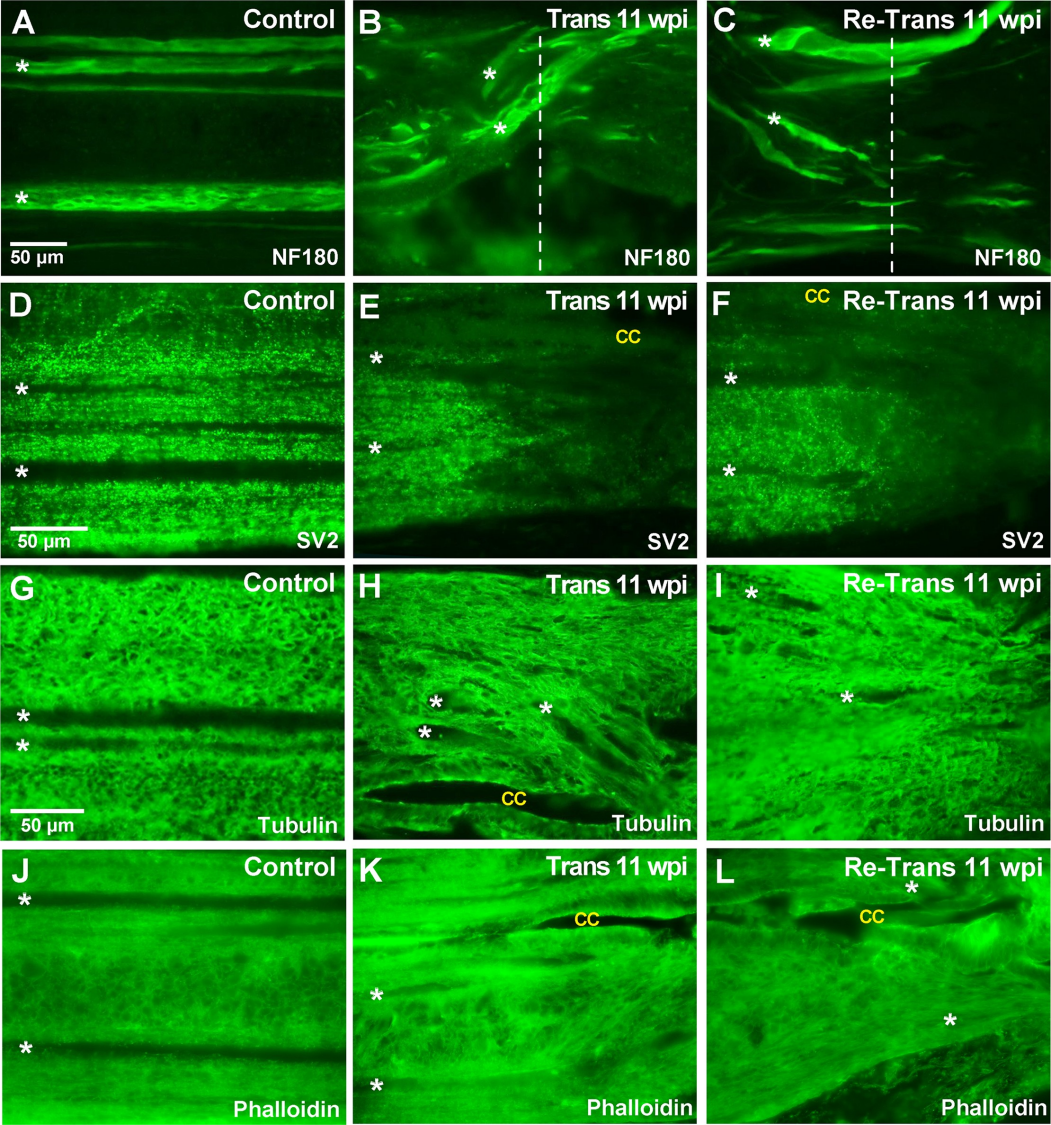
Cellular distribution of axons, synapses, and cytoskeleton are also similar at 11 wpi after spinal transection and re-transection. **(A-C)** NF-180 staining of large and medium caliber axons in the control, transected and re-transected spinal cords. Note the aberrant axonal growth patterns after spinal injury. Dotted lines indicate lesion center and show a number of regenerating axons. Scale bar in A applies to all panels except D and G. Asterisks indicate several large RS axons here and in all subsequent panels. **(D-F)** SV2 staining shows punctate labeling of synapses in all conditions. Note the tapering signal at the rostral-lesion border in the transected and re-transected spinal cords. CC = central canal. **(G-I)** Tubulin staining labels microtubule-rich processes throughout the neuropil. There are no major differences after spinal re-transection. **(J-L)** Phalloidin staining labels F-actin and appears as a diffuse signal surrounding axons and cell bodies in the spinal cord. Rostral is to the left in all images.

**Fig 5:**
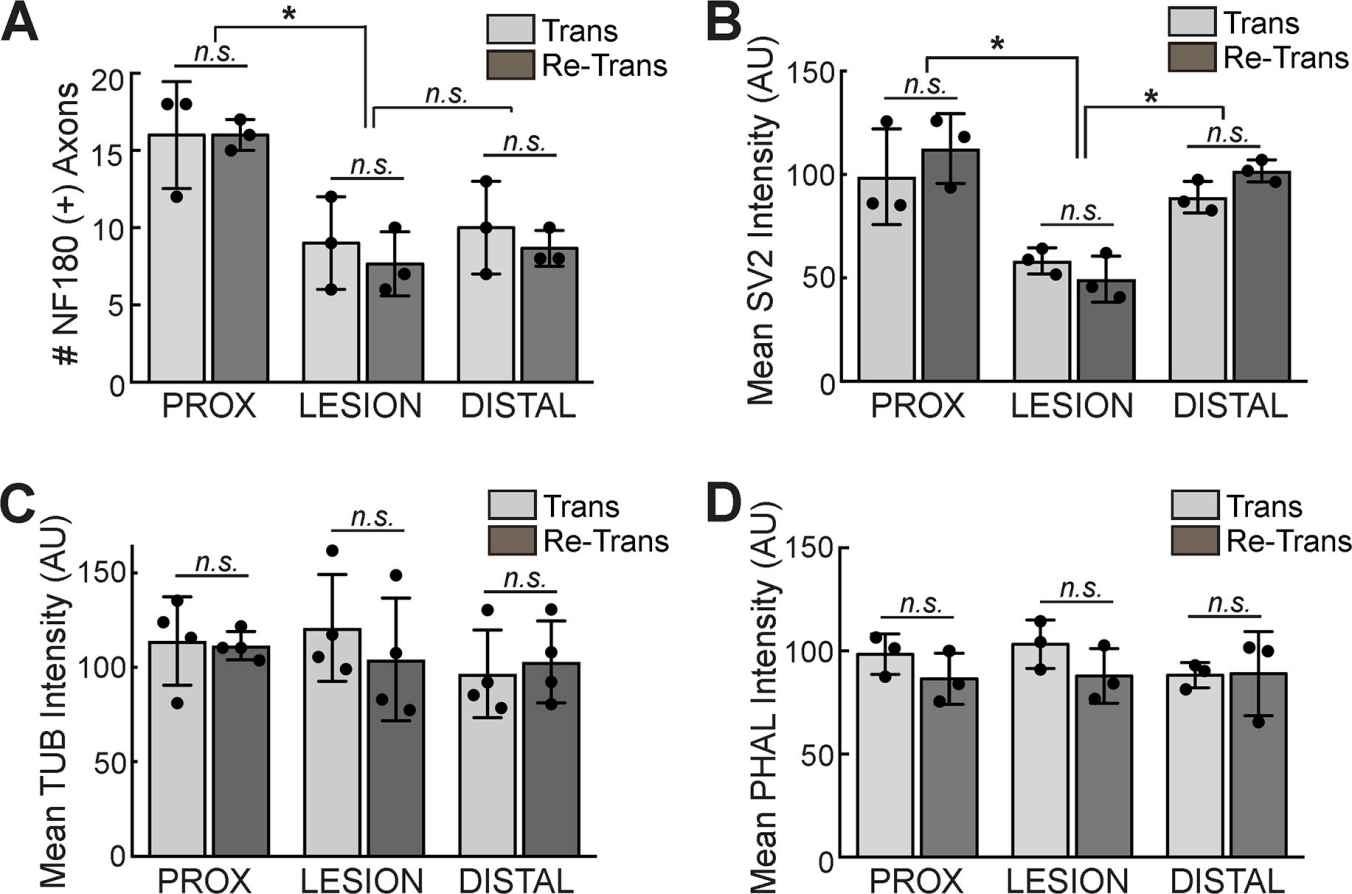
Quantification of NF-180, SV2, tubulin, and phalloidin staining reveals no significant differences after spinal re-transection. **(A)** Though there was a decrease in the number of NF-180 (+) axons within and distal to the lesion, compared to proximally, no differences were observed between transected and re-transected spinal cords at all locations. **(B)** Similarly there were no differences in the SV2 immunofluorescence intensity in transected and re-transected spinal cords, though the signal was reduced within the lesion. **(C-D)** Tubulin and phalloidin staining show similar levels of microtubule and F-actin throughout the spinal cord in transected and re-transected animals. AU = arbitrary units. Data represents mean ± SD from n = 3–4 experiments. * indicates p<0.05 by ANOVA. n.s. = not significant.

As another means to assess structural repair, we examined the distributions of several neuronal and cytoskeletal elements at 11 wpi after spinal transection or re-transection. Horizontal sections of lamprey spinal cords were prepared by cryosectioning. In order to ensure consistent evaluation of the same section plane, we focused on sections taken through the center of the central canal (Fig 3A), which occurs within a ventral region of the spinal cord and comprises predominantly motor tracts (i.e. those that drive swimming behaviors). The main anatomical features include large and medium caliber spinal axons, densely packed ependymal cells lining the central canal, and the somata of motor and intraspinal neurons (Fig 3B). In the transected spinal cord at 11 wpi, this section plane includes similar anatomical features, but with a swollen central canal and reduced numbers of axons (Fig 3C). We began by immunostaining sections with a mouse monoclonal antibody against lamprey neurofilament-180 (NF-180), which labels large and medium caliber axons in the lamprey spinal cord [53, 58]. In the control spinal cord, NF-180 immunostaining revealed a number of large RS axons extending in straight projections throughout the ventromedial and lateral tracts (Fig 3D). DAPI staining robustly labeled the nuclei of ependymal glial cells, which were densely packed around the central canal, as well as nuclei of motor and intraspinal neurons in the lateral columns (Fig 3D). At 11 wpi in the transected spinal cord, NF-180 labeling revealed some regenerating axons extending through the lesion site and into the distal stump, but with atypical projection patterns; DAPI labeling showed a central canal that was enlarged at the lesion site (Fig 3E). Similar patterns for NF-180 and DAPI staining were observed in the re-transected spinal cord at 11 wpi (Fig 3F). At higher magnification, the altered axonal growth patterns can be seen more clearly. Whereas most large RS axons were straight in the uninjured control spinal cord, the axons in transected and re-transected spinal cords instead projected in winding paths as they crossed through the lesion site (Fig 4A–4C). We performed a quantitative analysis on the number of NF-180 positive axons in three independent regions of the spinal cord: proximal, within, and distal to the lesion center. This analysis revealed that while the number of NF-180 (+) axons was significantly reduced within and distal to the lesion site, there was no difference between the transected and re-transected conditions at all locations tested (Fig 5A) (Proximal -Trans: 16.0 ± 3.5 axons; Re-Trans: 16.0 ± 1.0 axons; Lesion—Trans: 9.0 ± 3.0 axons; Re-Trans: 7.7 ± 2.1 axons; Distal: Trans: 10.0 ± 3.0 axons; Re-Trans: 8.7 ± 1.2 axons; n = 3 spinal cords/condition; One-way ANOVA p = 0.0028). The reduction in labeled axons beyond the lesion site is consistent with previous reports that only ~50% of descending axons regenerate [41–42, 44, 47]

Next, lamprey spinal cord sections were immunolabeled with an antibody against the synaptic vesicle glycoprotein SV2, which labels presynaptic vesicle clusters in all vertebrates tested, including lampreys [49, 57, 59, 60]. This allowed us to determine the overall distribution of synapses within the spinal cord. In the uninjured control spinal cord, the SV2 antibody produced fairly uniform, punctate staining throughout the neuropil (Figs 3G and 4D). In this view, the profiles of giant RS axons were visible as dark lines with little to no labeling, because their synapses are localized to the periphery along the axolemmal surface. In the transected spinal cord at 11 wpi, the density of synapses remained high in the proximal and distal regions, but was markedly reduced within the lesion site (Fig 3H), as previously reported [41]. A similar loss of SV2 labeling at the lesion site was also seen within re-transected spinal cords (Fig 3I). Higher magnification imaging revealed the decline in SV2 expression at the rostral-lesion border within transected and re-transected spinal cords (Fig 4E and 4F). Quantification corroborated the significant reduction in SV2 staining, and thus synapses, within the lesion site, but with no significant differences between transected and re-transected spinal cords (Fig 5B) (Proximal -Trans: 99 ± 23 AU; Re-Trans: 113 ± 17 AU; Lesion—Trans: 58 ± 6 AU; Re-Trans: 49 ± 11 AU; Distal—Trans: 89 ± 7 AU; Re-Trans: 102 ± 5 AU; n = 3 spinal cords/condition; One-way ANOVA p = 0.0004).

We also immunostained for α-tubulin and phalloidin, which label microtubules and filamentous actin, respectively. The tubulin antibody was a mouse monoclonal that recognized a single band in both rat brain and lamprey CNS lysates at ~50 kDa, which is the expected molecular weight for tubulin (S4 Fig). Both the α-tubulin and phalloidin staining were robust and relatively uniform throughout control spinal cords, except for the giant RS axons, which expressed lower levels of these cytoskeletal elements (Fig 3J and 3M). At higher magnification, tubulin staining revealed web-like structures throughout the neuropil (Fig 4G), which are intertwining processes of intraspinal neurons and glial cells, while phalloidin staining was more diffuse throughout the neuropil (Fig 4J). Similar distributions were observed for microtubules and F-actin after spinal transection and re-transection, albeit with some disorganization due to the tissue repair at the lesion site (Fig 3K, 3L, 3N and 3O and Fig 4H, 4I, 4K and 4L). These observations were corroborated by quantitative analyses, which showed no significant differences between the tubulin staining in transected and re-transected spinal cords (Fig 5C) (Proximal -Trans: 114 ± 23 AU; Re-Trans: 112 ± 7 AU; Lesion—Trans: 121 ± 28 AU; Re-Trans: 104 ± 33 AU; Distal—Trans: 97 ± 23 AU; Re-Trans: 103 ± 22 AU; n = 4 spinal cords/condition; One-way ANOVA p = 0.75). Neither were there any differences in the phalloidin staining (Fig 5D) (Proximal -Trans: 99 ± 10 AU; Re-Trans: 87 ± 12 AU; Lesion—Trans: 103 ± 12 AU; Re-Trans: 88 ± 13 AU; Distal—Trans: 88 ± 6 AU; Re-Trans: 89 ± 20 AU; n = 3 spinal cords/condition; One-way ANOVA p = 0.55). Taken together, these data indicate that the distributions of axons, synapses, and cytoskeletal elements within lamprey spinal cords are similar after two bouts of spinal repair and regeneration.

### Long-distance axon regeneration remains robust after spinal re-transection

**Fig 6:**
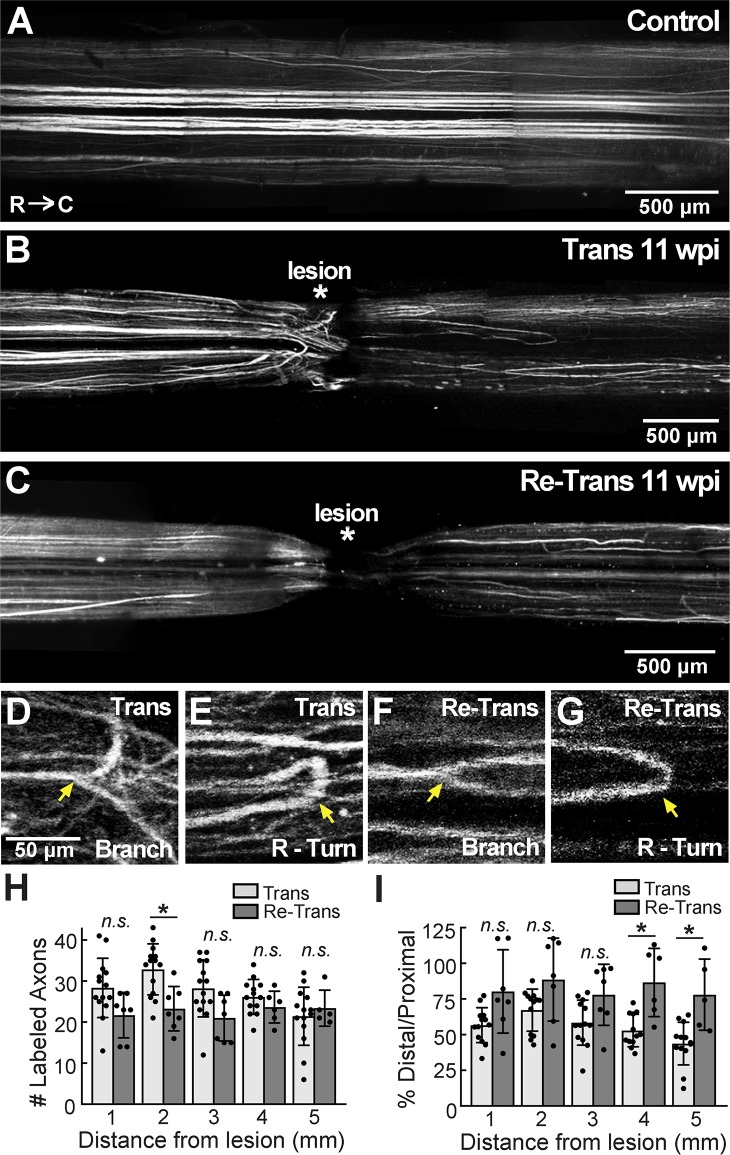
Axon regeneration in the lamprey spinal cord is comparable after repeated transections. **(A)** A montage of confocal z-projections showing bulk labeled axons in the uninjured, control spinal cord. Axons were labeled with 10 kDa Alexa-Fluor 488 dextran. Note the straight axonal projection patterns. (**B-C)** In contrast, at 11 wpi regenerated axons in the transected and re-transected spinal cords were sparser, and they exhibited atypical projection patterns in the medial and lateral tracts. **(D-G)** Higher magnification confocal images showing axonal branching and rostral turning (R-Turn) in transected and re-transected spinal cords (arrows) at 11 wpi. Scale bar in D applies to E-G. Rostral is to the left in all images. **(H)** In general, the number of labeled, axons was similar in transected and re-transected spinal cords. Bars represent mean ± SD from n = 7–14 spinal cords. n.s. = not significant; * indicates p<0.05 by ANOVA. **(I)** The percentage of distal/proximal labeled axons was slightly higher in re-transected spinal cords. Bars represent mean ± SD from n = 7–14 spinal cords. n.s. = not significant; * indicates p<0.05 by ANOVA.

Next, we examined the extent of axon regeneration after spinal cord injury. To do so, we anterogradely labeled regenerating axons in transected and re-transected lamprey spinal cords using a 10 kDa Alexa Fluor 488-conjugated fluorescent dextran, as previously described [51]. Within the uninjured control spinal cord, this procedure preferentially labeled giant RS axons in the ventromedial tract of the spinal cord, as well as medium-caliber fibers in the ventromedial and lateral tracts, the vast majority of which exhibited straight projection patterns (Fig 6A). In contrast, labeled axons within the transected spinal cord exhibited atypical projection patterns at 11 wpi, including branching and rostral turning, which was especially prevalent proximal to the lesion site (Fig 6B, 6D and 6E), as previously reported [41, 46, 47]. Regenerated axon branches in the distal spinal cord also had smaller diameters (Fig 6B). Similar morphologies and axonal growth patterns were observed in the re-transected spinal cords at 11 wpi (Fig 6C, 6F and 6G). The mechanisms that induce axonal branching and turning are unclear. However, it has been postulated that the altered morphologies of regenerated axons in the lamprey spinal cord are caused by changes in the expression levels of several axon guidance molecules, including increased expression of semaphorin 3 (a chemorepellent) and decreased expression of netrins (a chemoattractant), which consequently may lead to failure of some axons to regenerate [55, 62]. As a semi-quantitative approach to measuring axon regeneration, we counted the number of labeled axons crossing at 1 mm intervals beyond the lesion center up to 5 mm distal [51]. This analysis showed little difference in the number of labeled, regenerated axons in transected versus re-transected spinal cords at each distance measured (Fig 6H) (n = 14 Trans, 7 Re-Trans; One-way ANOVA, p<0.0001; Tukey’s post hoc analysis on Trans vs. Re-Trans at: 1 mm, p = 0.3644; 2 mm, p = 0.0326; 3 mm, p = 0.2526; 4 mm, p = 0.9980; 5 mm, p = 0.9998). Previous studies in the lamprey model have reported that ~50% of descending RS axons regenerate to a position distal to the lesion by 11 wpi [42, 44, 47, 50]. Thus, as an estimate for axon regeneration (and to normalize for any differences in overall labeling), we also calculated the percentage of labeled distal/proximal axons and observed similar values ranging from ~40–60% in the singly transected spinal cords with somewhat higher values in the re-transected spinal cords, (Fig 6I) (n = 14 Trans, 7 Re-Trans; One-way ANOVA, p<0.0001; Tukey’s post hoc analysis on Trans vs. Re-Trans at: 1 mm, p = 0.1862; 2 mm, p = 0.3101; 3 mm, p = 0.4408; 4 mm, p = 0.0168; 5 mm, p = 0.0245). Thus, it appears that axonal regrowth was not impaired by spinal re-transection but remained as robust or perhaps slightly better than after single spinal transections.

### Regeneration of giant RS neurons was similar after spinal transection and re-transection

**Fig 7:**
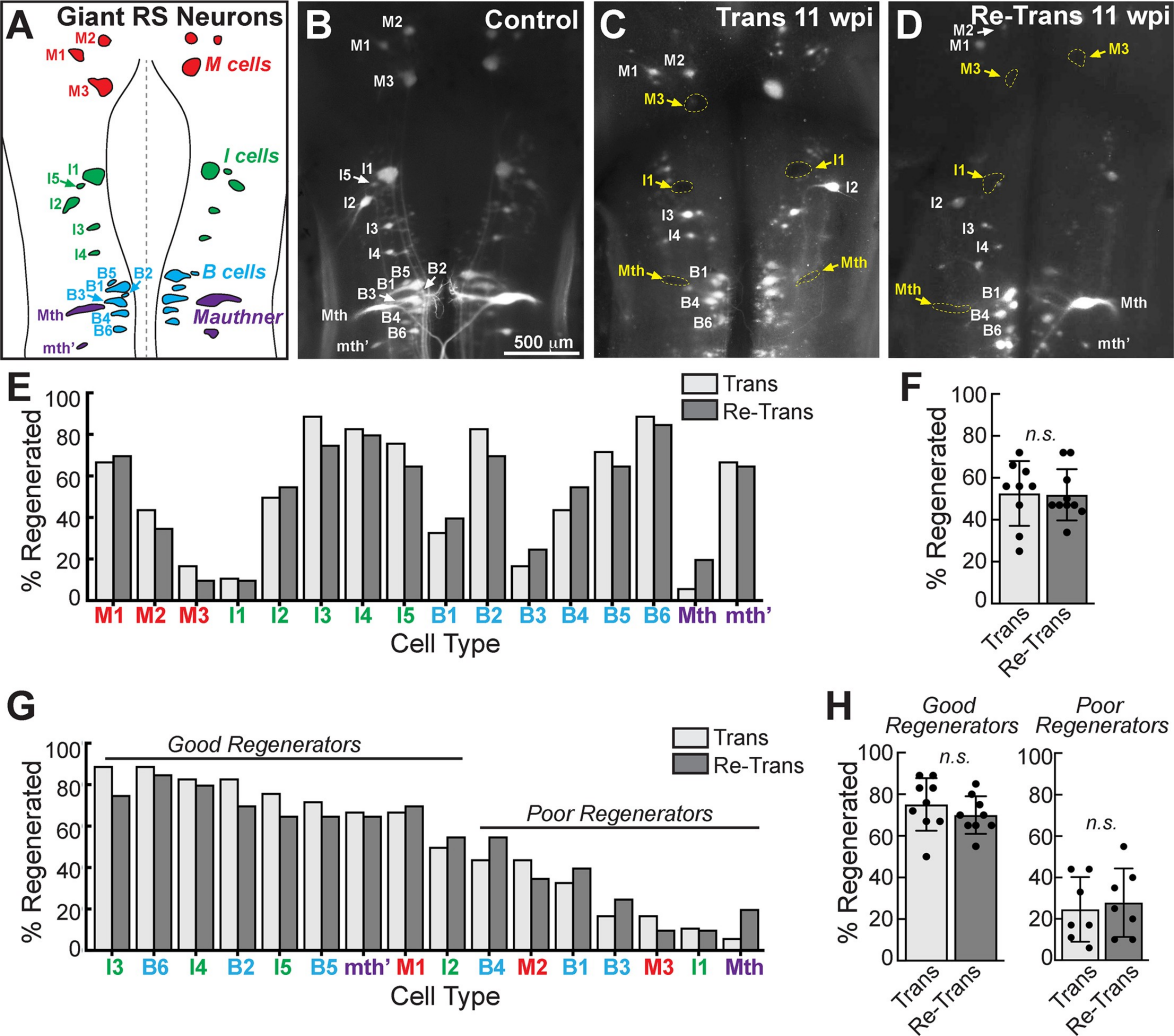
Regeneration of giant RS neurons is indistinguishable at 11 wpi after spinal transection and re-transection. **(A)** Diagram showing the giant RS neurons in the lamprey brain. These are the M, I, and B cells, as well as Mauthner neurons. **(B)** Retrograde labeling using tetramethylrhodamine dextran (10 kDa) in a control lamprey reveals all giant RS neurons. **(C)** In contrast, only a subset of giant RS neurons regenerate by 11 wpi after spinal transection (white labels), as identified by dye labeling, while others fail to regenerate and are therefore devoid of dye (yellow labels). **(D)** A similar cohort of regenerated neurons is labeled at 11 wpi after spinal re-transection. Scale bar in B also applies to C-D. **(E)** Cell-by-cell analysis of giant RS neuron regeneration from a population of n = 10 lampreys. There are no obvious differences in regeneration by cell type. **(F)** Similarly, the percentage of all giant RS neurons that regenerated was similar in transected and re-transected lampreys. **(G-H)** Likewise, there were no obvious differences in regeneration of either “good regenerators” or “poor regenerators.” Bars in F and H represent mean ± SD per brain from n = 10 lampreys. n.s. indicates “not significant” by unpaired Students t-Test (p>0.05).

We next took advantage of the large, identified giant RS neurons as another means to evaluate axon regeneration and neuronal survival after spinal re-transection. The lamprey midbrain and hindbrain contain ~1200 total RS neurons that descend into the spinal cord and initiate locomotion [63]. Amongst them are 32 identified giant neurons (100–200 μm in diameter) that reside in stereotypical locations (Fig 7A) [49, 50, 52, 64]. These include the mesencephalic cells (M cells: M1-3), isthmic cells (I cells: I1-I5), and bulbar cells (B cells: B1-B6), as well as the Mauthner (Mth) and auxiliary Mauthner (mth’) neurons. These giant RS neurons exhibit distinct intrinsic capacities for surviving and regenerating after axotomy induced by spinal cord transection [48–53]. While some giant RS neurons are “good regenerators” (e.g. M1, I3-I5, B2, B4-B6, mth’), meaning they have a high probability of surviving the injury and regenerating their axons, others are “poor regenerators” (e.g. M2-M3, I1, B1, B3, Mth) with a low probability of survival and regeneration.

We thus wanted to determine whether the giant RS axons retain their normal intrinsic capacities for regeneration after spinal re-transection. To do so, we retrogradely labeled regenerating RS neurons with tetramethylrhodamine applied caudal to the original lesion site (*see* Methods) [52]. In the brains of uninjured control animals, all giant RS neurons were labeled using this technique (Fig 7B). At 11 wpi after a single spinal transection, a select subset of giant RS neurons was labeled, indicating that they had regenerated their axons beyond the lesion site, and these were generally those neurons previously classified as “good regenerators” (Fig 7C, white labels). In contrast, the remaining giant RS neurons were not labeled, indicating that they did not regenerate their axons, and these were generally those neurons previously classified as “poor regenerators” (Fig 7C, yellow arrows). A similar pattern of RS neuron labeling was observed in the brains of re-transected lampreys, implicating a similar degree of neuron regeneration (Fig 7D). A cell-by-cell analysis was performed on a population of n = 9–10 lamprey brains. Since there are only 2 neurons of each type per brain, we present the summary population data for each cell type, as in our previous studies [49, 53]. Indeed, this cell-by-cell analysis revealed a similar degree of axon regeneration across each of the giant RS neurons after spinal transection or re-transection (Fig 7E). Across the entire population of 32 giant RS neurons, there was a similar degree of axon regeneration per brain at 11 wpi in transected or re-transected animals (Fig 7F) (Trans: 52.6 ± 15.5% regenerated neurons/brain, n = 9 animals, 288 neurons; Re-Trans: 51.9 ± 12.2% regenerated neurons/brain, n = 10 animals, 320 neurons; unpaired Student’s t-Test, p = 0.92). Likewise, at 11 wpi after transection or re-transection, there was a similar degree of regeneration amongst the “good regenerator” population (i.e. M1, I2-5, B2, B5-6, mth’), which we defined as those giant RS neurons that regenerated >50% of the time (Fig 7G and 7H) (*Good Regenerators*—Trans: 75.1 ± 12.7% regenerated neurons/brain, n = 9 animals, 162 neurons; Re-Trans: 70.0 ± 9.0% regenerated neurons/brain, n = 10 animals, 180 neurons; unpaired Student’s t-Test, p = 0.34). There was also a similar amount of regeneration amongst the “poor regenerators” (i.e. M2-3, I1, B1, B3-4, Mth), which we defined as those giant RS neurons that regenerated <50% of the time (Fig 7H) (*Poor Regenerators*—Trans: 24.6 ± 15.7% regenerated neurons/brain, n = 9 animals, 108 neurons; Re-Trans: 27.9 ± 16.6% regenerated neurons/brain, n = 10 animals, 120 neurons; unpaired Student’s t-Test, p = 0.71). Of interest is that several “poor regenerators” appeared to regenerate slightly better after spinal re-transection (e.g. B1, B3, Mauthner), though this was not significant across the larger neuronal population. Thus, the extent of giant RS axon regeneration observed after spinal re-transection was comparable to that after single transections, with the same population of “good regenerators” exhibiting robust regrowth.

### Nissl staining of giant RS neurons was similar after spinal transection and re-transection

As with axon regeneration, each giant RS neuron exhibits a distinct and reproducible intrinsic capacity for survival or death after injury-induced axotomy. The “good survivors” (e.g. M1, I2-5, B2, B6, mth’) are those giant RS neurons that typically survive the injury and regenerate their axons, while the “poor survivors” (e.g. M3, I1, B1, B3, Mth) are those that typically undergo delayed death by apoptosis [49, 52, 53, 65].

**Fig 8:**
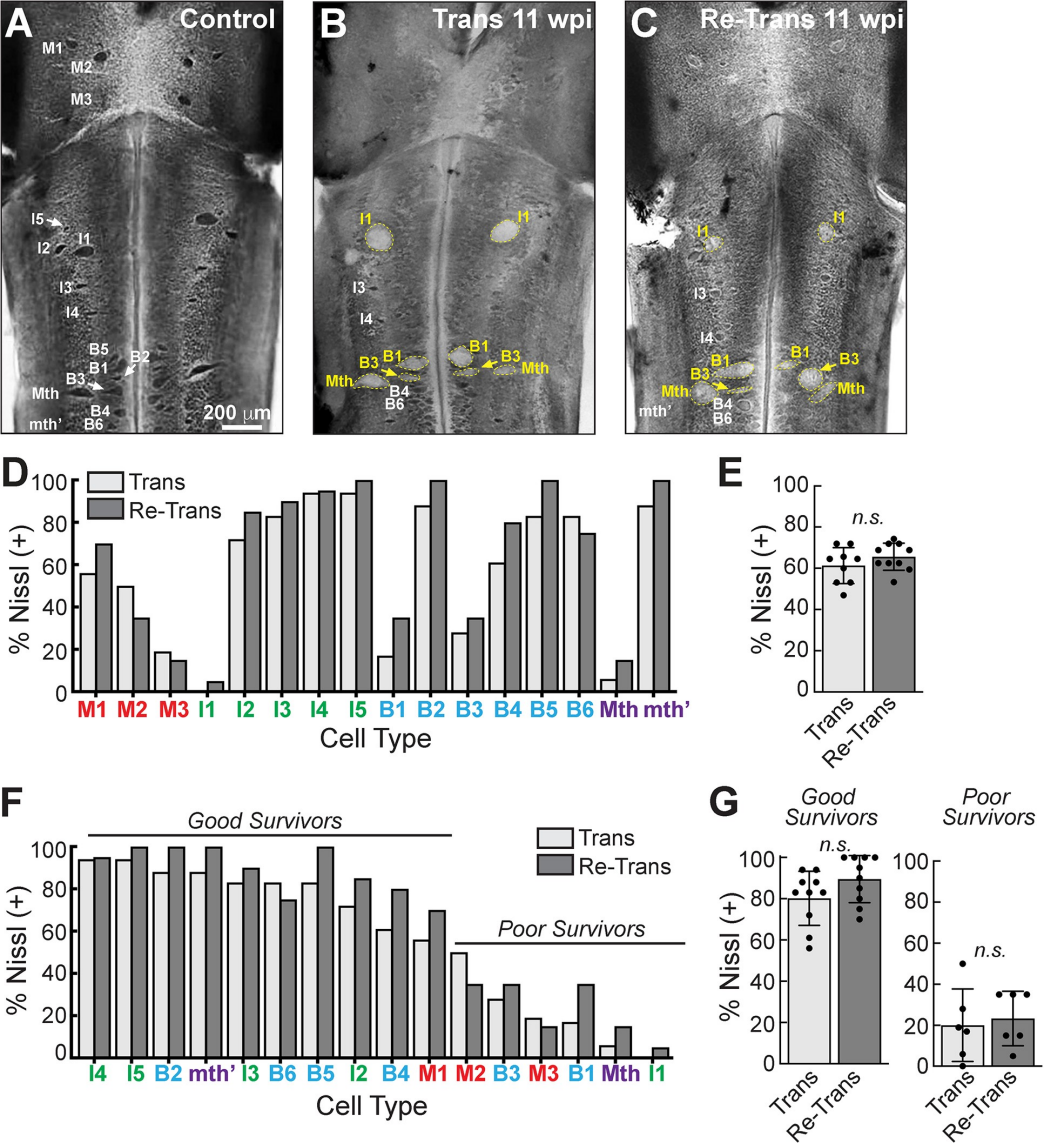
Nissl staining of giant RS neurons is comparable at 11 wpi after spinal transection and re-transection. **(A)** A whole mounted control lamprey brain stained with Toluidine blue O, which labels Nissl substance within healthy neurons. All giant RS neurons are labeled. Scale bar in A also applies to B-C. **(B-C)** In contrast, at 11 wpi after spinal transection and re-transection only a subset of neurons retains strong Nissl staining (white labels), indicating surviving neurons. Other neurons become chromalytic, swell, and lose their Nissl substance (yellow labels), indicating neurodegeneration. **(D)** Cell-by-cell analysis of Nissl (+) giant RS neurons from n = 10 lampreys. **(E)** The percentage of total giant RS neurons that was Nissl (+) was similar in transected and re-transected lampreys. **(F-G)** “Good survivor” and “poor survivor” populations of giant RS neurons exhibited similar degrees of Nissl (+) staining. Bars in E and G represent mean ± SD per brain from n = 10 lampreys. n.s. indicates “not significant” by Students t-Test (p>0.05).

As a second measure of giant RS neuron vitality, we evaluated cell survival and death in the same brains that had been previously assayed for axon regeneration [49, 52, 65]. After live imaging to assess the regenerated RS neurons, as described in Fig 7, we then fixed and histologically stained the brains with Toluidine blue O, which labels Nissl substance. All giant RS neurons within control brains were darkly stained, revealing abundant Nissl substance that is characteristic of healthy neurons (Fig 8A). We refer to these as “Nissl (+)” neurons. In contrast, at 11 wpi after spinal transection, a subset of neurons (largely the “poor survivors”) had a swollen, chromalytic appearance and lacked Nissl substance, which is indicative of degenerating neurons (Fig 8B, yellow labels) [49, 52]. We refer to these as “Nissl (-)” neurons. The remaining neurons (largely “good survivors”) retained Nissl substance after spinal transection (Fig 8B). At 11 wpi after spinal re-transection, a similar Nissl (+) staining pattern was observed amongst the giant RS neurons (Fig 8C). When quantifying the data on a cell-by-cell basis, it appeared that a greater percentage of many giant RS neurons was Nissl (+) stained after spinal re-transection (Fig 8D and 8F). This included neurons identified as “good survivors”, which we defined as those that retained Nissl (+) staining >50% of the time (i.e. M1, I2-I5, B2, B4-6, mth’), as well as “poor survivors”, which we defined as those giant RS neurons that retained Nissl (+) staining <50% of the time (i.e. M2-3, I1, B1, B3, Mth) (Fig 8F). However, when quantified across the entire population of 32 giant RS neurons, there was no statistically significant difference in the percentage of total Nissl (+) giant RS neurons per brain at 11 wpi after spinal re-transection (Fig 8E) [Trans: 61.3 ± 8.7% Nissl (+) neurons/brain, n = 9 animals, 288 neurons; Re-Trans: 65.6 ± 6.6% Nissl (+) neurons/brain, n = 10 animals, 320 neurons; unpaired Student’s t-Test, p = 0.24]. Likewise, there was no significant difference in the percentage of Nissl (+) “good survivors” per brain after spinal re-transection (Fig 8G) (*Good Survivors*: Trans: 80.2 ± 13.1% Nissl (+)/brain, n = 9 animals, 180 neurons; Re-Trans: 89.5 ± 11.4% Nissl (+)/brain; n = 10 animals, 200 neurons; unpaired Students t-Test, p = 0.11). Nor were there differences in the percentage of Nissl (+) “poor survivors” (Fig 8G) [*Poor Survivors*: Trans: 20.0 ± 17.7% Nissl (+)/brain, n = 9 animals, 108 neurons; Re-Trans: 23.3 ± 13.3% Nissl (+)/brain, n = 10 animals, 120 neurons; unpaired Student’s t-Test, p = 0.72]. Thus, the probability of survival amongst all giant RS neurons was similar in transected and re-transected lampreys, and “good survivors” appeared to robustly survive the second injury.

### The relationship between giant RS neuron regeneration and survival was unaltered after spinal re-transection

**Fig 9:**
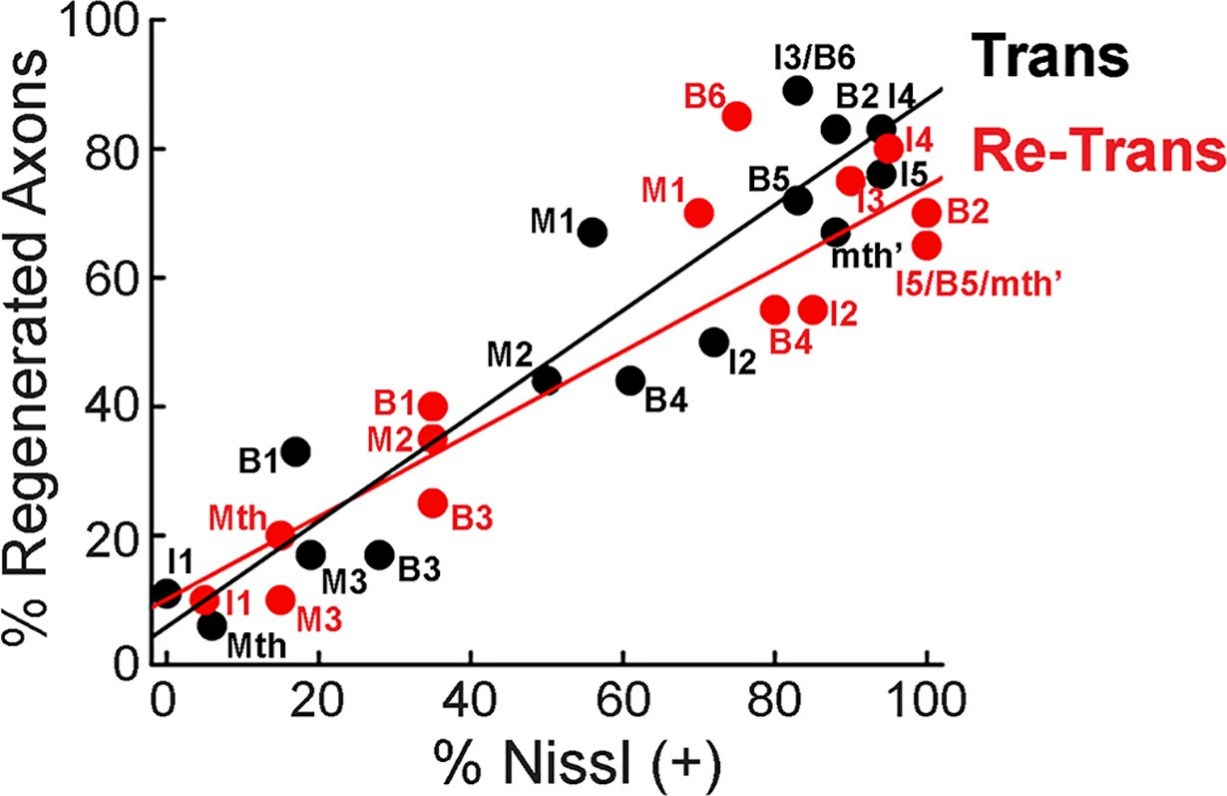
The relationship between cell survival and axon regeneration is similar at 11 wpi after spinal re-transection. There is a positive, linear correlation between cell survival [Nissl (+)] and axon regeneration after spinal transection (R^2^ = 0.91), which is also recapitulated after spinal re-transection (R^2^ = 0.84) (ANCOVA p = 0.11).

Previous studies have reported a positive linear correlation between neuronal survival (as measured by Nissl staining) and axon regeneration (as measured by retrograde labeling) for the giant RS neurons, such that neurons with a high probability of survival at 11 wpi are also likely to regenerate their axons and vice versa [48–50, 52]. Using this same approach, we also observed the same positive correlation at 11 wpi after a single spinal transection, as was previously reported (Fig 9, black line; R^2^ = 0.91). The same strong correlation was also observed after spinal re-transection (Fig 9, red line, R^2^ = 0.84), which was not significantly different from the former (One-way ANCOVA, p = 0.11). Thus, at 11 wpi after both spinal cord transection and re-transection, the relationship between neuronal survival and axon regeneration was maintained for each of the giant RS neurons, further corroborating the sustained regenerative potential within lamprey spinal cord.

## Discussion

We report here that the regenerative capacity within the lamprey spinal cord appears to be largely unaltered after two successive transections at the same lesion plane. Behavioral recovery (Fig 1), tissue repair (Fig 2), synapse and cytoskeletal distributions (Figs 3–5), axon regeneration (Figs 6 and 7), and cell survival (Fig 8) were nearly identical after recovery from both the first and second spinal transections. Similarly, in axolotls, the area of tail tissue that regenerates after a second amputation is on average the same as after the first amputation, though differences between sexes have been observed [8]. The Japanese newt (*Cynops pyrrhogaster*) can regenerate a normal lens successively up to 18 times spanning 16 years, demonstrating a seemingly unlimited regenerative capacity that is also unaffected by aging [66]. Another striking example is the zebrafish caudal fin, which appears to exhibit unlimited regeneration by regrowing normal fin structures even after 27 amputations [35]. These instances are in stark contrast to repeated limb amputations at the same plane in amphibians such as axolotls and newts, which exhibit high fidelity regeneration after the first amputation but dramatically decreased regeneration with each successive injury, starting with the second amputation [36, 37]. Imperfect limb regeneration in amphibians has also been reported in the fossil record [38], though naturally the prior status of the limbs cannot be ascertained. Interestingly, in axolotls, performing limb amputations at serially-distal locations resulted in significantly improved regenerative capacity, indicating that the failure of limb regeneration is due to events occurring at the original lesion plane, which included aberrant collagen deposition [36]. We do not yet understand the full regenerative capacity in the lamprey spinal cord, which would require additional rounds of transection and regeneration. However, the robust, high fidelity regeneration that we observed after two successive spinal cord transections suggests that lampreys have greater regenerative capacity than is observed in some other highly regenerative models.

Functional recovery after spinal cord injury in lampreys and other non-mammalian vertebrates is supported by extensive regeneration of descending axons beyond the lesion scar [21, 26]. Previous studies in lampreys reported that ~50% of descending reticulospinal axons regenerated several millimeters beyond the lesion center by 11 wpi [41, 44, 47, 51]. Results presented here are consistent with this overall level of axon regeneration in the re-transected lamprey spinal cords after the second round of regeneration (Figs 6I and 7F). The percentages of regenerated axons appears to be slightly higher after bulk anterograde labeling (Fig 6I), but this may be due to the possibility of counting multiple branches of the same parent axon that cross at the fiduciary marker.

Remarkably, the cell specificity of axon regeneration amongst the giant RS neurons was also maintained after spinal re-transection (Figs 7–9). On one hand, it is not surprising that “poor regenerators/survivors” did not regenerate after spinal re-transection, because they had likely undergone delayed degeneration by apoptosis after the first spinal transection, as previously reported [48, 49, 52, 53, 65]. However, it is interesting that the extent of axon regeneration and cell survival of the remaining RS neurons, that is the “good regenerators/survivors” (e.g. M1, I2-I5, B2, B5-B6, mth’), was nearly the same after both spinal transection and re-transection (Figs 7–9). It is unlikely that these giant RS neurons were replaced by newly-born neurons, as neurogenesis appears to be fairly limited in the brain after spinal injury and also restricted to the ependymal zone [67]. It is thus likely that many of the “good regenerators/survivors” underwent two rounds of regeneration during the 22-week experiment, suggesting that the intrinsic regenerative capacity of individual giant RS neurons was also largely unaffected by spinal re-transection. Determining this unequivocally would require long-term dynamic imaging in the lamprey nervous system, which is not yet practical in our model but is under development.

While lampreys recover normal swimming behaviors after spinal cord transection and re-transection, it must be acknowledged that functional recovery is the result of substantial plasticity throughout the lamprey CNS. That is, the regenerated spinal cord does not return to the original status of an uninjured spinal cord but rather forms new functional circuitry with compensatory network properties [68]. This is clearly illustrated by the facts that only a subset of descending axons regenerate in the transected and re-transected spinal cord (Figs 6 and 7), and of those that regenerate many terminate early and exhibit atypical projection patterns (Fig 6), and produce few synapses (Figs 3–5) [41, 46, 47, 69]. Yet, the excitatory postsynaptic potentials, a measure of synaptic strength, can be as strong or stronger than those in the uninjured spinal cord [45, 70]. In addition, using electrophysiological methods, compensatory plasticity has also been documented at regenerated synapses in the ventral spinal cord of lampreys, as are changes in the intrinsic properties of regenerated axons, which together could boost the synaptic output of regenerated synapses [68, 71]. Alterations in the expression levels of axon guidance molecules, cell proliferation/death genes, ion channels, immune system, and various other neuronal genes have also been reported after spinal cord injury in lampreys [55, 62, 72], as have expression changes in multiple neurotransmitter systems [73–76], implicating another level of molecular plasticity that underlies successful regeneration in this model. Given the remarkable consistency of axon, synapse and cytoskeleton distributions (Figs 3–5); axon regeneration (Figs 6 and 7); and cell survival (Fig 8) in the transected and re-transected spinal cords, it is likely that the second bout of regeneration induces similar types of molecular, anatomical, and physiological plasticity, though this remains to be fully explored. Likewise, it will be important to study regeneration mechanisms in the dorsal tracts of the re-transected spinal cords, which carry predominantly sensory information.

Even though spinal cord repair and regeneration remained robust after repeated injuries, there were also some notable differences in the re-transected spinal cords. For example, at 1 wpi the re-transected spinal cords appeared to exhibit accelerated tissue repair (Fig 2 and S1 Fig). And at 3 and 11 wpi, the re-transected spinal cords were narrower at the lesion center (Fig 2; S2 and S3 Fig). Despite this narrowing of the lesion site, the re-transected spinal cords still exhibited normal or slightly higher numbers of regenerated axons (Figs 6 and 7) and typical restoration of swimming behaviors (Fig 1). Thus, although there were differences in several gross anatomical features of the re-transected spinal cords, suggesting somewhat different mechanisms, nonetheless these differences did not alter the degree of functional recovery.

Going forward, it will be important to further investigate the cellular and molecular mechanisms of tissue repair and regeneration after spinal re-transection. Doing so would allow us to identify how regenerative capacity remains so robust after additional injuries. RNA-Seq analysis, such as that which was recently performed on singly transected spinal cords [55], may therefore be useful as an unbiased means for beginning to identify these mechanisms in the re-transected spinal cords. Doing so will permit a greater understanding of the molecular requirements that are driving successful regeneration of the vertebrate CNS and may provide insights into the limitations that occur in non-regenerative models such as the mammalian CNS.

## Supporting information

S1 Appendix Excel spreadsheet containing raw data from the study. Each sheet contains the individual data points used in a particular figure, as noted.
(XLSX)

S1 Fig Transected and re-transected lamprey spinal cords at 1 wpi. **A.** Bright field images showing lamprey spinal cords at 1 wpi after the initial transection. Note the large gap between the proximal and distal stumps. **B.** In contrast, at 1 wpi after spinal re-transection, the gap between the stumps appears smaller. In all images, the arrow indicates the central canal. Asterisks indicate the lesion center. Red box indicates the image shown in the main Fig 2. Scale bar applies to all images.
(TIFF)

S2 Fig Transected and re-transected lamprey spinal cords at 3 wpi. **A.** Bright field images showing lamprey spinal cords at 3 wpi after the initial transection. The lesion site is now repaired, and no gap exists between the stumps. **B.** At 3 wpi after spinal re-transection, the lesion is also repaired but appears narrower. In all images, the arrow indicates the central canal. Asterisks indicate the lesion center. Red box indicates the image shown in the main Fig 2. Scale bar applies to all images.
(TIFF)

S3 Fig Transected and re-transected lamprey spinal cords at 11 wpi. **A.** Bright field images showing lamprey spinal cords at 11 wpi after the initial transection. The spinal cord appears more repaired and has regained its translucency. **B.** At 11 wpi after spinal re-transection, the spinal cord appears similar but remains narrower. In all images, the arrow indicates the central canal. Asterisks indicate the lesion site. Red box indicates the image shown in the main Fig 2. All scale bars = 500 nm.
(TIFF)

S4 Fig Characterization of the α-tubulin antibody. Western blot using a mouse monoclonal α-tubulin antibody (Sigma; clone DM1A) revealed a single band in both rat brain and lamprey CNS lysates at ~50 kDa, which is the expected molecular weight for α-tubulin.
(TIFF)
