# Supplementary figures and images for: Regenerative capacity in the lamprey spinal cord is not altered after a repeated transection
Source: PLoS One. 2019 Jan 30;14(1):e0204193. doi: 10.1371/journal.pone.0204193 (PMC6353069; doi:10.1371/journal.pone.0204193)

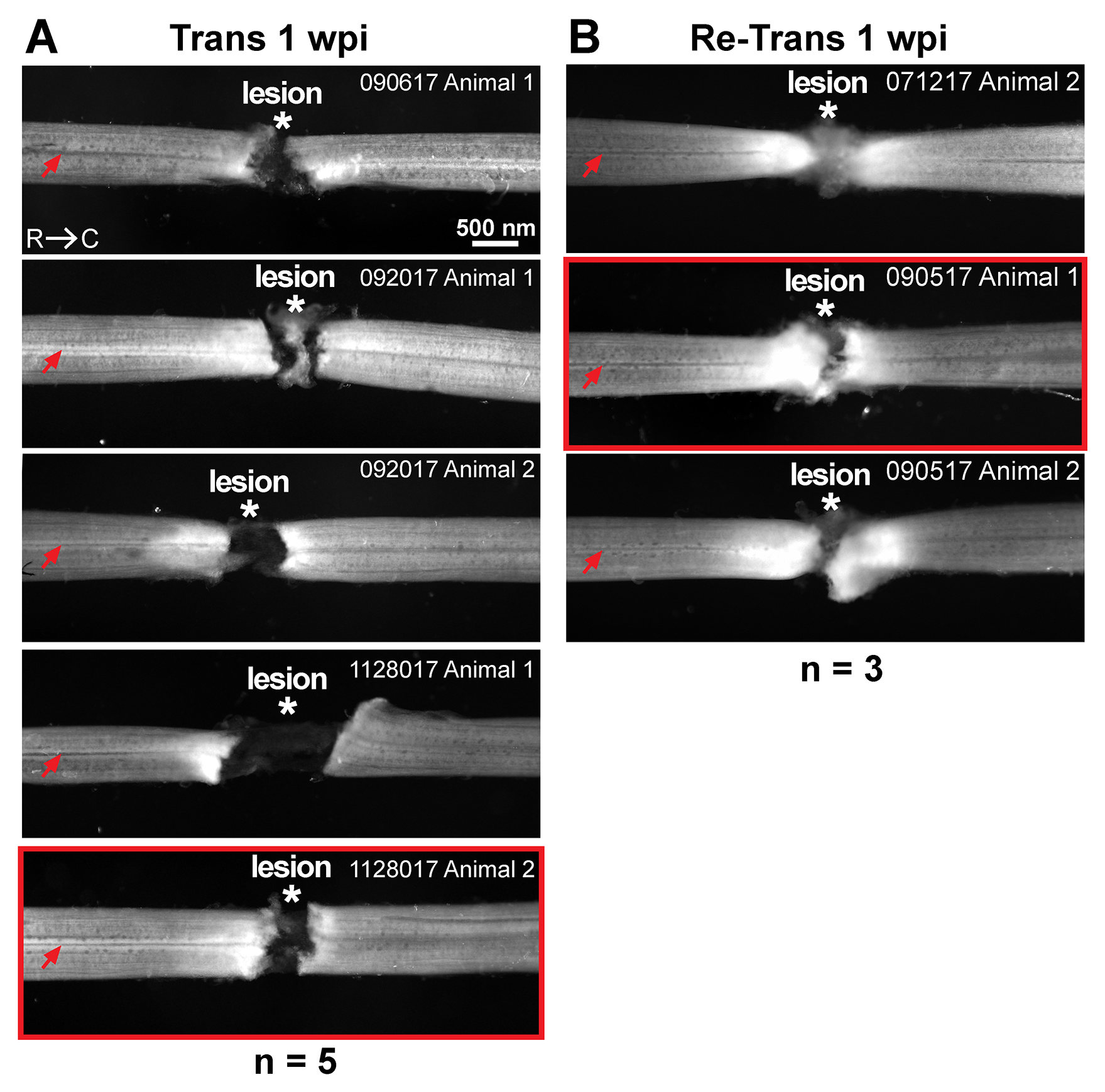

Supplement: S1 Fig — A. Bright field images showing lamprey spinal cords at 1 wpi after the initial transection. Note the large gap between the proximal and distal stumps. B. In contrast, at 1 wpi after spinal re-transection, the gap between the stumps appears smaller. In all images, the arrow indicates the central canal. Asterisks indicate the lesion center. Red box indicates the image shown in the main Fig 2. Scale bar applies to all images. (TIFF) [file pone.0204193.s002.tiff]

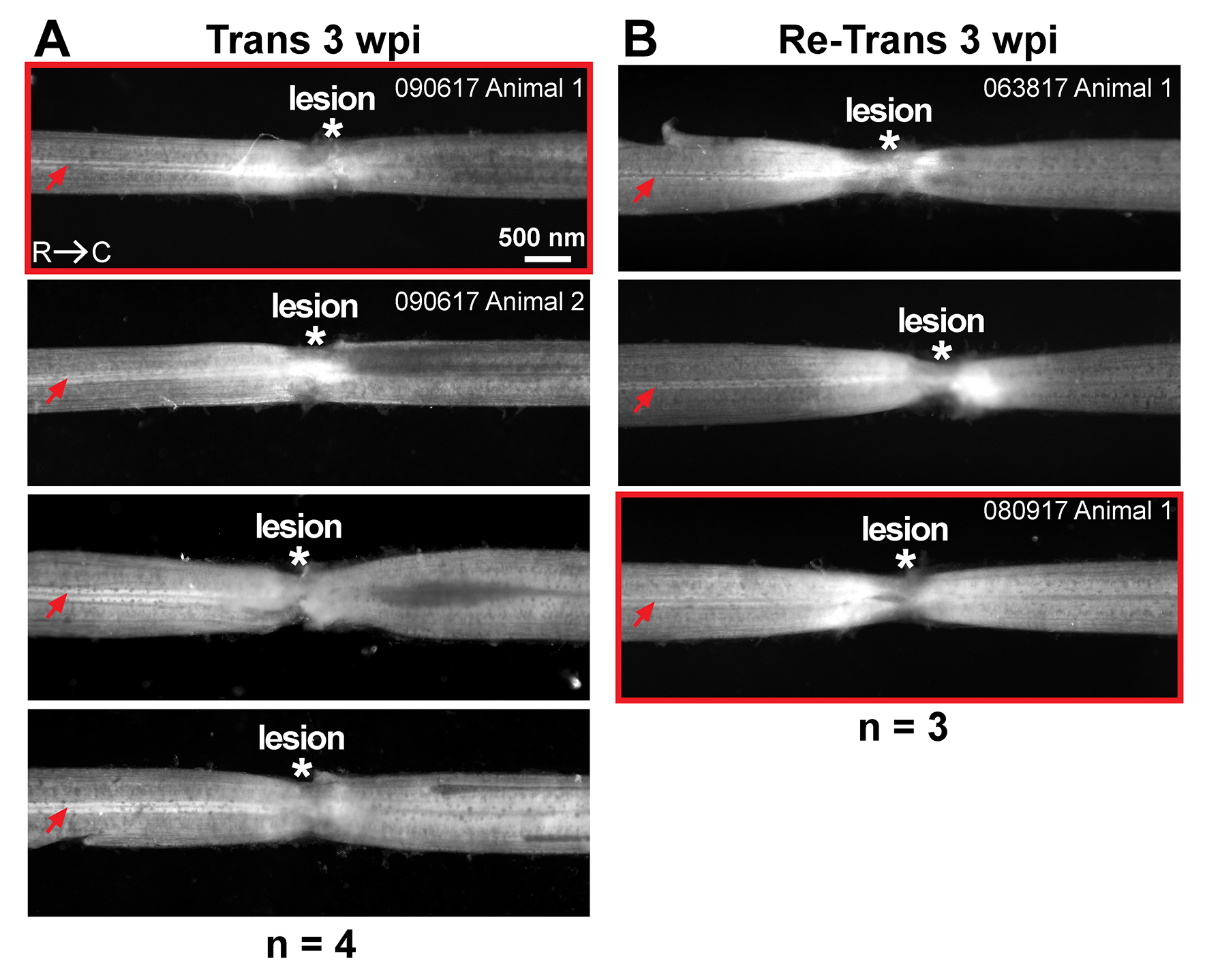

Supplement: S2 Fig — A. Bright field images showing lamprey spinal cords at 3 wpi after the initial transection. The lesion site is now repaired, and no gap exists between the stumps. B. At 3 wpi after spinal re-transection, the lesion is also repaired but appears narrower. In all images, the arrow indicates the central canal. Asterisks indicate the lesion center. Red box indicates the image shown in the main Fig 2. Scale bar applies to all images. (TIFF) [file pone.0204193.s003.tiff]

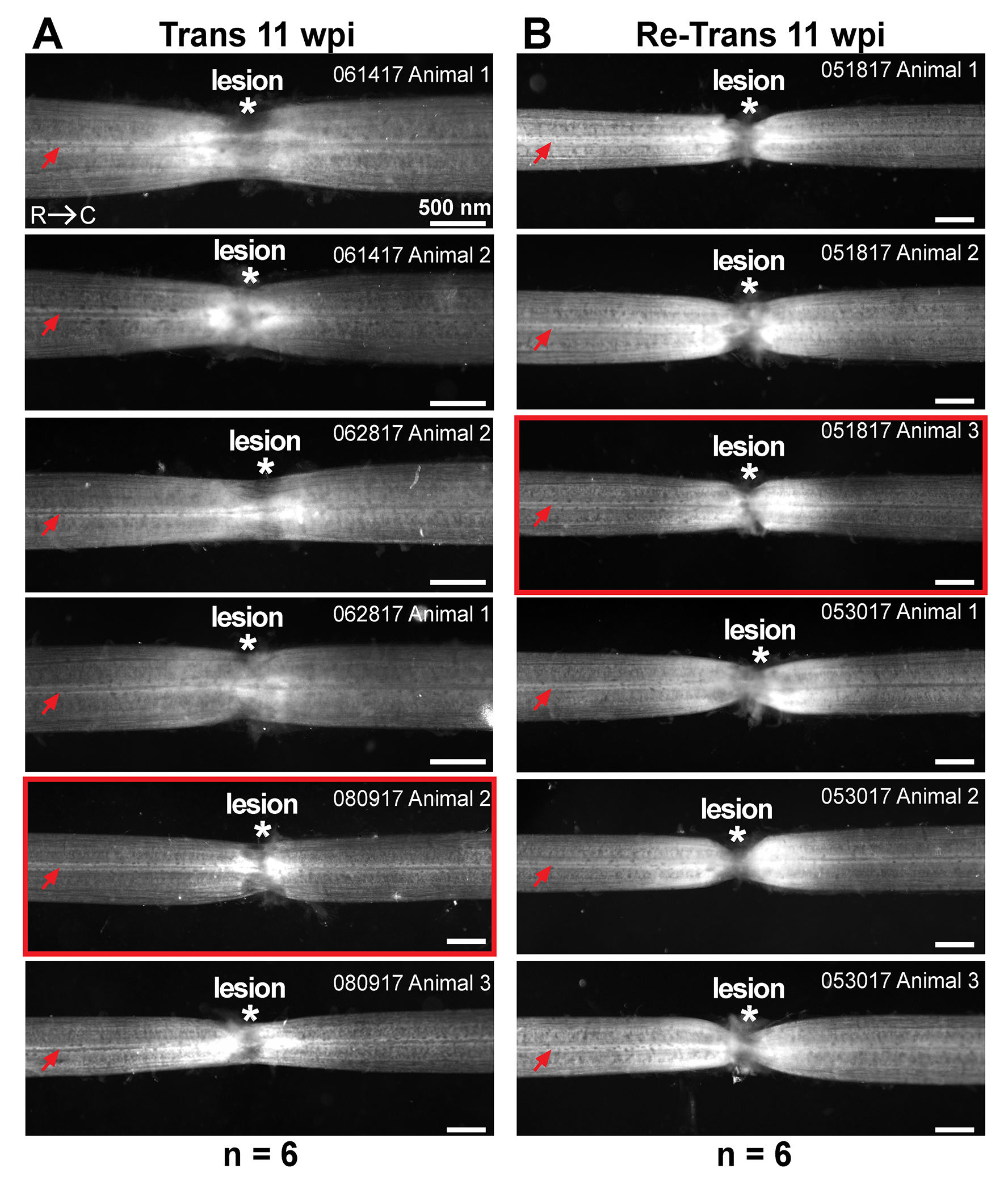

Supplement: S3 Fig — A. Bright field images showing lamprey spinal cords at 11 wpi after the initial transection. The spinal cord appears more repaired and has regained its translucency. B. At 11 wpi after spinal re-transection, the spinal cord appears similar but remains narrower. In all images, the arrow indicates the central canal. Asterisks indicate the lesion site. Red box indicates the image shown in the main Fig 2. All scale bars = 500 nm. (TIFF) [file pone.0204193.s004.tiff]

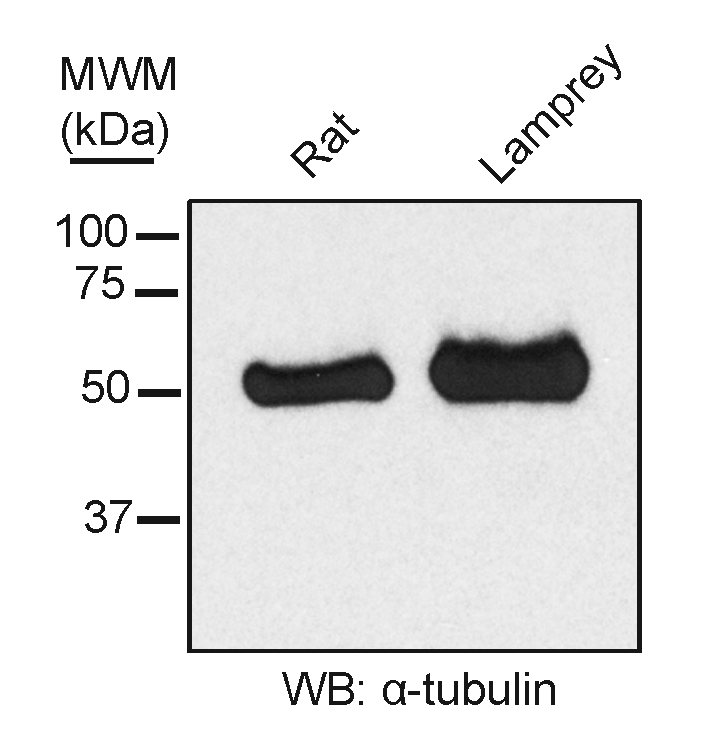

Supplement: S4 Fig — Western blot using a mouse monoclonal α-tubulin antibody (Sigma; clone DM1A) revealed a single band in both rat brain and lamprey CNS lysates at ~50 kDa, which is the expected molecular weight for α-tubulin. (TIFF) [file pone.0204193.s005.tiff]
